# Recent Progress in Regulating the Activity of Enzymes with Photoswitchable Inhibitors

**DOI:** 10.3390/molecules29194523

**Published:** 2024-09-24

**Authors:** Yi Chen

**Affiliations:** 1 Key Laboratory of Photochemical Conversion and Optoelectronic Materials, Technical Institute of Physics and Chemistry, Chinese Academy of Sciences, Beijing 100190, China; yichen@mail.ipc.ac.cn; 2 School of Future Technology, University of Chinese Academy of Sciences, Beijing 100190, China

**Keywords:** photopharmacology, photoswitchable inhibitor, enzyme activity, azobenzene unit

## Abstract

Photoregulation of biomolecules has become crucial tools in chemical biology, because light enables access under mild conditions and with delicate spatiotemporal control. The control of enzyme activity in a reversible way is a challenge. To achieve it, a facile approach is to use photoswitchable inhibitors. This review highlights recent progress in photoswitchable inhibitors based on azobenzenes units. The progress suggests that the incorporation of an azobenzene unit to a known inhibitor is an effective method for preparing a photoswitchable inhibitor, and with these photoswitchable inhibitors, the activity of enzymes can be regulated by optical control, which is valuable in both basic science and therapeutic applications.

## 1. Introduction

The activity of an enzyme plays an important role in many physiological, pathological, and pharmacological processes, and the abnormal activity of an enzyme is directly related to various cancers [1,2,3,4,5]. The inhibition of enzymes activity is often performed with their corresponding inhibitors. However, the limitation of many inhibitors is that they cannot control the location and duration of their inhibitory activity. Thus, strategies for targeting the activity of an enzyme are of great interest for both therapeutic and basic science applications [6,7,8,9,10,11,12,13].

Light is unsurpassed in its ability to control biological systems with high spatial and temporal resolution in a non-invasive manner. Furthermore, light wavelength and intensity can be precisely regulated and does not cause contamination of the sample [14]. Recently, photopharmacology that combines photochemistry and pharmacology has grown considerably and shows its advantages in living systems [15,16,17,18,19,20,21]. By functionalizing biomolecules with molecular photoswitches, light-sensitive and switchable biomolecules can be obtained. These photoswitchable biomolecules are introduced into some important biological processes to regulate fast and reversibly biological activities. This approach has been used to photoregulate a multitude of important biological processes, including DNA/RNA [22,23,24,25], proteins [26,27,28], enzymes [29,30,31], ion channels [32,33,34], transporters [35,36,37], receptors [38,39,40], and others [41,42,43,44]. This review summarizes recent progress in the development of photoswitchable inhibitors for enzymes activity regulation, specifically the use of azobenzenes as photoswitch scaffolds. The irreversible regulation, photoswitchable inhibitors based on other photoswitch scaffold or photoswitchable inhibitors used for photoregulation of other biological processes are beyond the scope of this review.

## 2. Design Strategy of the Photoswitchable Inhibitor and Its Regulation Mechanism

The activity of enzymes is affected by enzyme inhibitors, and the properties of inhibitors depend on their structures and conformation. A candidate photoswitchable inhibitor shall have some characteristics, (1) easy switching between on and off states upon irradiation with a given wavelength, (2) significant difference in inhibition effect between on and off states, and (3) facile preparation.

**Scheme 1:**
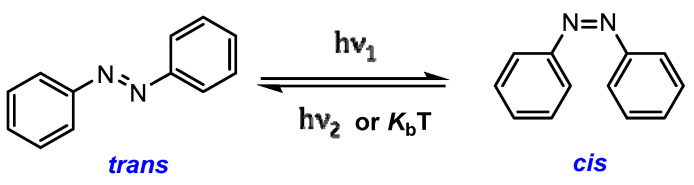
Photoisomerization of azobenzene. hν_1_ and hν_2_ stand for light to switch; *k*_b_T stands for thermal relaxation.

Azobenzene (Scheme 1) has been widely utilized as a photoswitch due to its easy accessibility, small size, and good optical properties [45,46]. In response to different irradiations, azobenzene isomerization between *trans*- and *cis*-configurations is accompanied by large, reversible changes in molecular geometry and polarity, resulting in distinct pharmacological properties [47]. Therefore, incorporating an azobenzene unit into a known inhibitor to produce a photoswitchable inhibitor is a simple and efficient method.

**Scheme 2:**
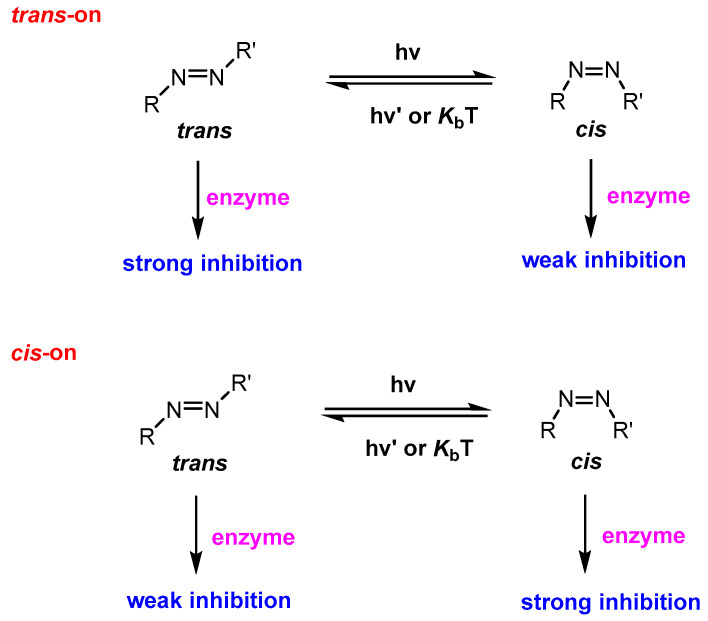
Diagram of photoswitchable inhibitors with *trans*-on and *cis*-on mechanisms, respectively.

Generally, there are two mechanisms (Scheme 2) for regulating the activity of enzymes with photoswitchable inhibitors. One is the “*trans*-on” mechanism, in which the *trans* of a photoswtichable inhibitor shows strong inhibition due to the strong interaction between inhibitor and target. Upon irradiation, the *trans* converts into its *cis*, and the *cis* exhibits weakened or relieved inhibition, since there is no or a slight interaction between inhibitor and target owing to a distinguished change in configuration. The other is the “*cis*-on” mechanism, in which *trans* exhibits weak inhibition due to its structure mismatching a target. The inhibition is, however, significantly increased when *trans* converts into *cis* upon irradiation. For practical application, the latter is desired, because it can be initially inactive and active after light-triggering. For ideal applications of photopharmacology, initially inactive molecules should be instantly and dramatically activated by photoirradiation to exert their biological effects.

## 3. Photoregulating Activity of Enzymes

### 3.1. Trans-On Inhibitors

**Scheme 3:**
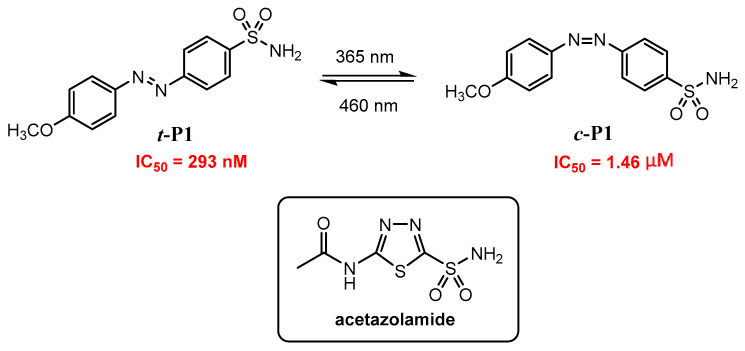
Photoisomerization of **P1** and the corresponding parent inhibitor.

Carbonic anhydrases are involved in various physiological and pathological processes and are regarded as important therapeutic targets, since they are overexpressed in many diseases, such as obesity, cancer, and epilepsy [48]. Aggarwal and co-workers [49] reported a *trans*-on photoswitchable inhibitor for the in situ photoregulation of carbonic anhydrase (CA) activity. A photoswitchable inhibitor **P1** (Scheme 3) was designed by the introduction of acetazolamide (CA inhibitor) into an azobenzene derivative. In the *trans*, ***t*-P1** is a linear shape that benefited from occupying the CA active site and interacting with Zn^2+^ via benzenesulfonamide, resulting in the inhibition of enzyme activity. Upon irradiation with 365 nm light, the ***t*-P1** isomerized to its *cis*, ***c*-P1**. The ***c*-P1** exited the active site due to the change in the steric profile, resulting in the restoration of enzyme activity. The ***c*-P1** can revert back to the ***t*-P1** through thermal relaxation or via photoirradiation with 460 nm light, thereby inhibiting enzyme activity again. In the photostationary state (PPS), 93% ***c*-P1** (at PPS 365 nm) and 70% ***t*-P1** (at PPS 460 nm) were obtained, respectively.

The inhibitory activity of **P1** with bCA was determined using the *p*-nitrophenyl acetate (NPA) hydrolysis assay [50]. The inhibitory effect showed that ***t*-P1** was five-fold more potent than the ***c*-P1**, with IC_50_ values of 293 nM and 1.46 µM, respectively. Furthermore, the inhibitory effect of **P1** on the CO_2_ hydration activity of bCA was analyzed by using bromothymol blue as a pH indicator [51]. The kinetics of the change in the pH values demonstrated that the rate of CO_2_ hydration reaction decreased significantly when bCA was incubated with ***t*-P1**, indicating enzyme inhibition. When the bCA was incubated with ***c*-P1**, the rate of CO_2_ hydrolysis increased, indicating a weaker inhibitory effect.

**Figure 1:**
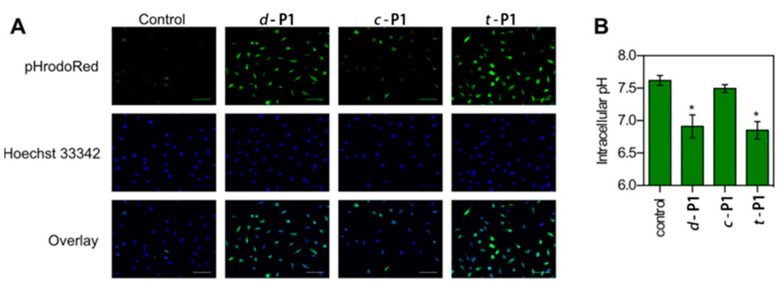
(**A**) Fluorescence imaging of pHrodo Red (green) as a result of different intracellular pH values of HeLa cells with different isomers of **P1** for 30 min. Blue fluorescence corresponds to a Hoechst stain. Scale bars are 100 μm. (**B**) Absolute pH values were calculated on the basis of a calibration curve and were reported as the mean of individual cells from three wells (N = 3; each replicate contains data from ~100 cells). Data represent mean values ± the standard deviation. Asterisks denote statistically significant differences (*p* = 0.05; one-way analysis of variance). Reproduced with permission from [49]. Copyright 2019 American Chemical Society.

The feasibility of **P1** to modulate the activity of cytosolic CAII in living cells was performed in HeLa cells. Cytosolic CAII serves as a driving force in cytosolic pH regulation [48]. The cells were incubated with a solution of ***d*-P1** (>98% *trans* isomer in the dark, which was irradiated with 365 and 460 nm light to generate ***c*-P1** and ***t*-P1**, respectively) and pHrodo Red AM (pH indicator that displays an increased fluorescence at a lower pH) for 30 min at 37 °C. As shown in Figure 1, cells treated with ***d*-P1** and ***t*-P1** displayed fluorescence values much higher than those of non-treated cells (control) and ***c*-P1**-treated cells. The corresponding pH values for these cells were 6.91 (***d*-P1**), 6.85 (***t*-P1**), 7.62 (control), and 7.50 (***c*-P1**). Both pH values of ***d*-P1**-treated cells and ***t*-P1**-treated cells are very close to acetazolamide (6.89) at the same concentration. The results indicated that ***t*-P1** showed more potential in pH regulation and CA inhibition compared to ***c*-P1**.

**Scheme 4:**
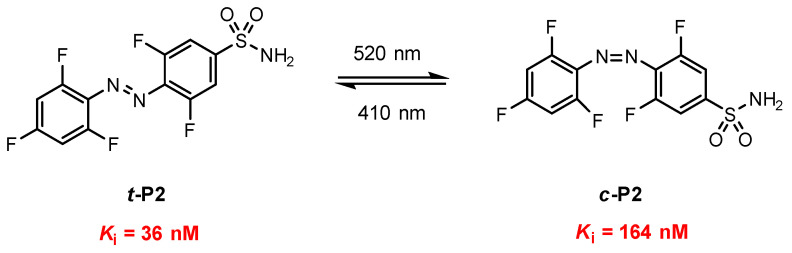
Photoisomerization of **P2**.

The same research group subsequently reported [52] another photoswitchable inhibitor, **P2** (Scheme 4), for CA activity regulation. The fluorinated azobenzenesulfonamide **P2** has some advantages of visible light isomerization and a more stable *cis* configuration. Upon irradiation with 520 nm, ***t*-P2** converted into *cis* with 87% of ***c*-P2** at PPS_520 nm_. Isomerization from *cis* to *trans* can subsequently be achieved with 410 nm light irradiation and with 82% of ***t*****-P2** at PPS_410 nm_.

Docking studies suggested that ***c*-P2** could not show any favorable interaction with Zn^2+^ in the active site, which made it bind more weakly than ***t*-P2**. The inhibitory effect of *trans* and *cis* of **P2** on the CO_2_ hydration activity of bCA was measured by tracking changes in the solution pH using the stopped flow method [53]. The apparent Ki values for ***t*-P2** and ***c*-P2** were calculated to be 36 ± 2 and 164 ± 8 nM, respectively. The results demonstrated ***t*-P2’s** great ability to inhibit CA enzymatic activity.

The real-time inhibition of cytosolic CA by ***t*-P2** was studied by the investigation of the rate of change of intracellular pH. It is known that the inhibition of cytoplasmic CA reduces the rate of intracellular acidification [54,55]. HeLa cells were loaded with pHrodo and incubated with 25 μM ***t*-P2**, ***c*-P2**, or dimethyl sulfoxide (DMSO) for the control. ***c*-P2** treated cells showed a similar rate of change in pH to the DMSO-treated control cells. The ***t*-P2** treated cells, however, displayed a slower rate of change in pH, which demonstrated inhibition of the cytosolic CA.

**Figure 2:**
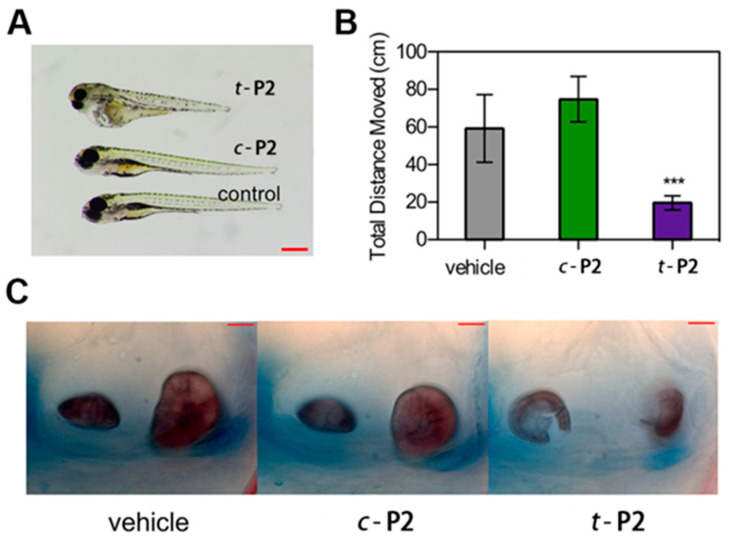
The morphological appearance (**A**), swimming behavior (**B**), and otolith development (**C**) of zebrafish in the absence (vehicle) and presence of ***t*-P2** and ***c*-P2**. The data represent the mean values ± the standard deviation. Asterisks denote statistically significant differences (*** *p* < 0.0001). Scale bars represent 500 and 50 μm for (**A**,**C**), respectively. Reproduced with permission from [52]. Copyright 2020 American Chemical Society.

In vivo evaluation of the regulation of enzyme activity by **P2** was carried out in zebrafish (wild-type embryos were collected and transferred into standard embryo media and sorted by developmental stage). It has reported that the inhibition of CA by inhibitors resulted in small otoliths, an irregular jaw, enlarged heart and yolk sac, and impaired locomotion [56,57]. As shown in Figure 2, the zebrafish treated with ***t*-P2** showed multiple morphological abnormalities, including failure to form a swim bladder, pectoral fin defects, and cardiac edema (Figure 2A), while the zebrafish treated with the same concentration of ***c*-P2** developed normally. Furthermore, the zebrafish treated with ***t*-P2** showed poor locomotion (Figure 2B) and had hollow and underdeveloped otoliths (Figure 2C), while ***c*-P2**-treated zebrafish exhibited normal locomotive behavior and normally developed otoliths (100%, n = 30).

**Scheme 5:**
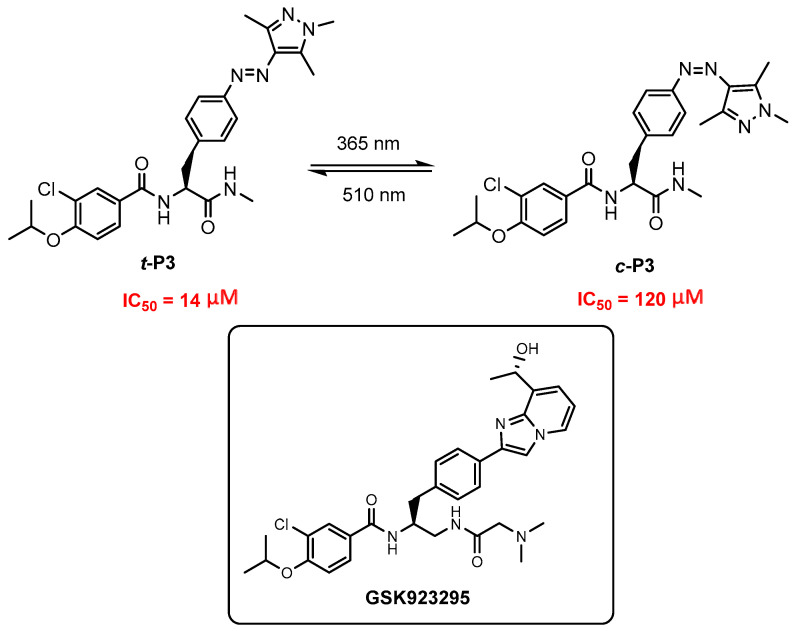
Photoisomerization of **P3** and the corresponding parent inhibitor.

Interfering with mitosis is a potential cancer therapy strategy. Nusrat Mafy and co-workers [58] developed a photoswitchable inhibitor for regulating the activity of centromere-associated protein E (CENP-E), a mitotic kinesin required for chromosome transportation. **P3** (Scheme 5) was designed based on GSK923295 [59], a CENP-E inhibitor. Replacing the core imidazopyridine ring in GSK923295 with azopyrazole provided photoswitchable inhibitor **P3**. Reversible *trans-cis* photoisomerization of **P3** was achieved upon irradiation with 365 nm and 510 nm light, respectively, with 93% of ***c*****-P3** at PSS_365nm_ and 86% of ***t*****-P3** at PSS_510 nm_.

GSK923295 is a selective CENP-E inhibitor and locks CENP-E by blocking inorganic phosphate release in its adenosine triphosphatease (ATPase) cycle [60,61]. The inhibitory effects of **P3** on ATPase activity showed large different IC_50_ values at PSS_510nm_ (14 μM) and at PSS_365nm_ (120 μM), which demonstrated that ***t*-P3** displayed ~10-fold inhibition activity as compared to ***c*-P3**. The similar results were obtained when **P3** was used for the inhibition of CENP-E activity in living cells. The inhibitory mechanism indicated that **P3** blocked CENP-E at the rigor state in living cells and could perturb chromosome congression in a photoswitchable manner.

**Figure 3:**
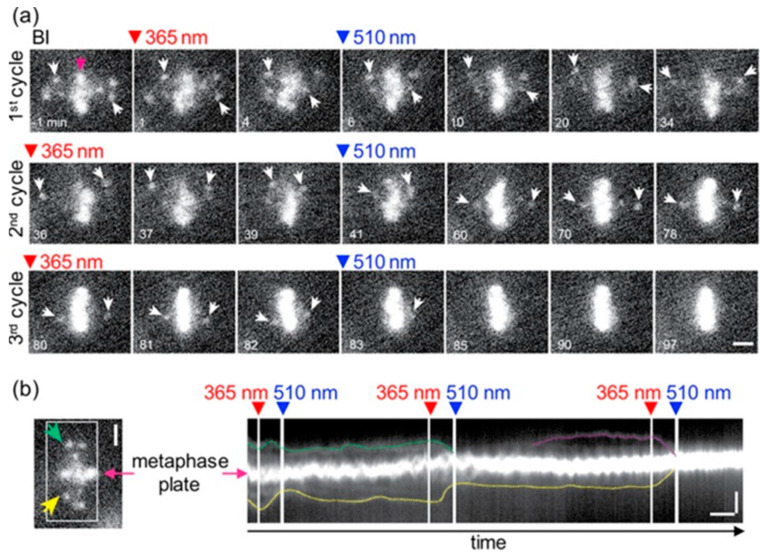
(**a**) Live imaging of mitotic chromosomes in a **P3**-treated LLC-PK1 cell under alternating 365 nm (red) and 510 nm (blue) light irradiation. White arrows: misaligned chromosomes; magenta arrow: aligned chromosomes. (**b**) A kymograph of mitotic chromosome movement along the cell division axis. The misaligned chromosomes are highlighted and tracked by lines with different colors. Vertical bars, 5 μm. Horizontal bar, 5 min. Reproduced with permission from [58]. Copyright 2020 American Chemical Society.

Photocontrollable mitotic interference was demonstrated by using **P3**-mediated chromosome congression in LLC-PK1 cells. As shown in Figure 3, the misaligned polar chromosomes, which were induced by pretreatment with **P3**, gradually moved toward the equatorial plane upon irradiation with 365 nm light (***t*-P3**). Subsequently, for irradiation with 510 nm light (***c*-P3**), the movement was ceased, and a portion of the misaligned chromosomes moved to the spindle poles. According to reports [59,62], GSK923295 induced frequent misalignment of chromosomes at the spindle poles; therefore, the obtained results demonstrated the inhibition of CENP-E by **P3** at PSS_510nm_. The direction of the chromosome movement changed repeatedly in the subsequent light irradiation cycle.

**Scheme 6:**
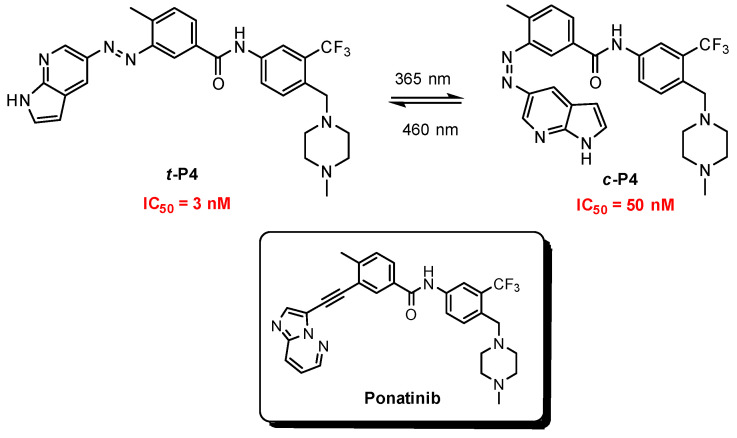
Photoisomerization of **P4** and the corresponding parent inhibitor.

REarranged during Transfection (RET) is a kinase belonging to the receptor tyrosine kinase family. Dysregulation of RET activity leads to several human cancers [63]. Xu and co-workers [64] reported a photoswitchable DFG-out kinase inhibitor (D: aspartic acid; F: phenylalanine; G: glycine), using RET as a model target. Photoswitchable inhibitor **P4** (Scheme 6) was designed by employing the known inhibitor Ponatinib [65] as a template. Docking studies suggested that the ***c*-P4** would not be tolerated in the active site, while the ***t*-P4** would serve as the active inhibitor. 

Irradiation with 365 nm light afforded trans→cis isomerization with 97% of ***c*-P4** at PPS_365nm_. The reverse reaction was achieved when exposed to blue light (460 nm), yielding 64% of ***t*-P4** at PPS_460nm_. Inhibitory studies showed that ***t*-P4** was 17-fold more potent than ***c*-P4**, with IC_50_ values of 3 nM and 50 nM, respectively. In vitro evaluation of **P4** was carried out by using nanobioluminescece resonance energy transfer (NanoBRET ™) determination of target engagement (TE) intracellular kinase in HEK293 cells, in which the IC_50_ values for ***t*-P4** and ***c*-P4** are 25 nM and 282 nM, respectively. 

**Scheme 7:**
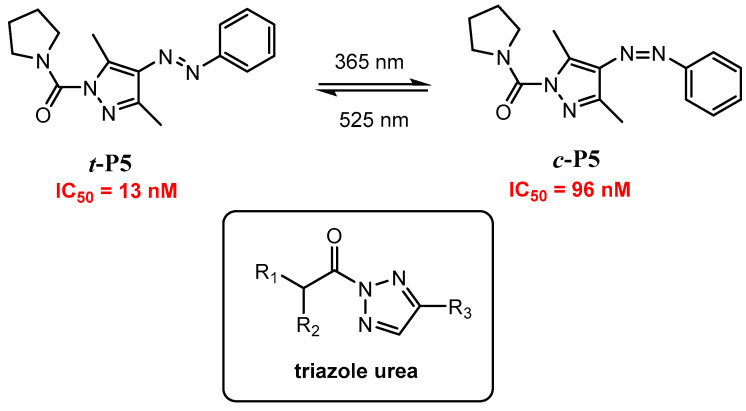
Photoisomerization of **P5** and the corresponding parent inhibitor.

Carboxylesterases (CES) are serine esterases from the alpha/beta-fold hydrolase family and can activate or deactivate therapeutics and affect the pharmacokinetics and the pharmacodynamics of the metabolized drugs by hydrolyzing ester and amide bonds of xenobiotics. Dwyer and co-workers [66] synthesized a class of arylazopyrazole urea-based photoswitchable inhibitors for human carboxylesterases 1 (CES1) and 2 (CES2). Among them, **P5** (Scheme 7) is the most promising candidate. **P5** was designed by modification of triazole ureas, serine hydrolase inhibitors [67], with photoswitchable arylazopyrazole. **P5** exhibited excellent conversion after 5 min of irradiation at room temperature, and 95% of ***c*-P5** was obtained at PSS_365nm_. The thermal relaxation of *cis*→trans was measured over time at 37 °C, and good thermal stability of ***c*-P5** was observed (t_1/2_ = 60 h).

Inhibitory studies were performed by employing CES1 as model, since CES1 is the best-studied xenobiotic ester hydrolytic enzyme in the human carboxylesterase family. CES1 inhibitory activity of ***t*-P5** and ***c*-P5** was measured using gel-based competition experiments [68] with a synthesized fluorescent probe. HepG2 cell lysate was treated with different concentrations of ***t*****-P5**, followed by treatment with the fluorescent probe, the CES1 band fluorescence signal was quantified, and IC_50_ values were calculated. It showed that ***t*-P5** was 7.4-fold more potent than ***c*-P5**, with IC_50_ values of 13 nM and 96 nM, respectively. Further study was conducted on live HepG2 cells. Cells were treated with ***t*-P5** and irradiated with 365 nm light for 5 min, then incubated for 4 h. Cells were lysed and treated with the fluorescent probe. The in situ treatment displayed a 5.1-fold difference in IC_50_ values of ***t*-P5** (5.0 nM) and ***c*****-P5** (25.4 nM). 

The evaluation of photoswitchable inhibitor **P5** in regulating CES1 activity was conducted by measuring the CES-catalyzed hydrolysis of mycophenolate mofetil (MMF). MMF is a known substrate of CES1 and an immunosuppressant that is widely applied to prevent organ transplant rejection and to treat Crohn’s disease [69]. It was found that ***t*-P5** inhibited CES1-mediated hydrolysis of MMF, and the half-life (t_1/2_) of MMF was increased to 79 min, whereas the t_1/2_ of MMF was decreased to 44 min with the ***c*-P5**-treated samples, which was nearly identical to the value obtained in the DMSO-treated samples (t_1/2_ = 43 min).

**Scheme 8:**
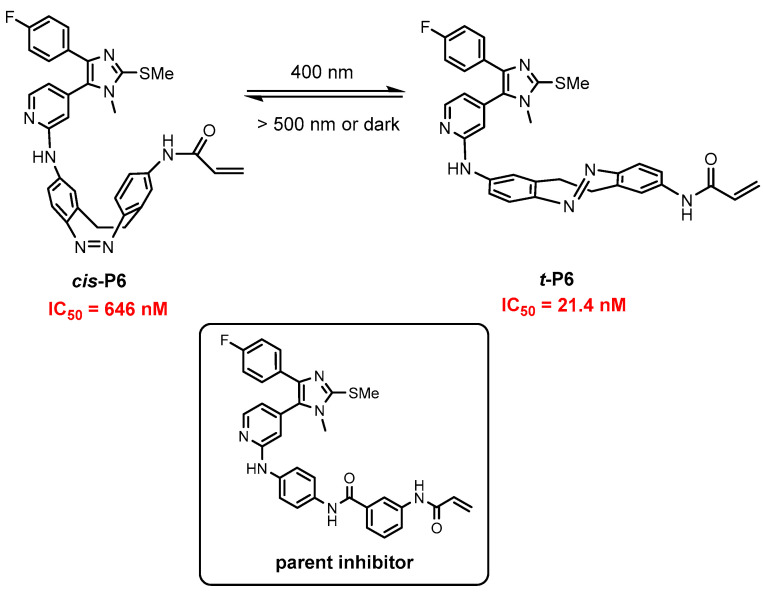
Photoisomerization of **P6** and the corresponding parent inhibitor.

The mitogen-activated protein (MAP) kinase c-Jun N-terminal kinase 3 (JNK3) is one of the key signaling enzymes in the cellular stress response and has been targeted for the treatment of neurodegenerative diseases, including Alzheimer’s and Parkinson’s disease [70]. Reynders and co-workers [71] developed a class of light-activated JNK3 inhibitors. Among them, inhibitor **P6** (Scheme 8) shows the most promising candidate for the inhibition of JNK3. **P6** was obtained by replacing the diarylamide motif of the known covalent inhibitor [72] with a diazocine photoswitch bearing an electrophilic acrylamide moiety that targets cysteines neighboring the ATP-binding site. Unlike other azobenzenes, ***c*-P6** is thermally stable. Molecular docking suggested that both ***c*-P6** and ***t*-P6** could bind to the ATP pocket of JNK3, but only the metastable ***t*-P6** could reach and covalently bind to Cys154.

The inhibition of **P6** to JNK3 was determined through measuring the phosphorylation of the immobilized kinase substrate activating transcription factor 2 (ATF-2) at different inhibitor concentrations. ***c*-P6** exhibited a weak inhibition to JNK3 (IC_50_ = 646 nM) but showed much stronger inhibition at all tested concentrations after pulse irradiation with 390 nm light (IC_50_ = 21.4 nM). It is worth noting that, once isomerization and potential covalent attachment took place, inhibition with ***t*-P6** was found to be irreversible, probably because binding to the ATP binding site was too tight to pull the pyridinylimidazole from the active site.

### 3.2. Cis-On Inhibitors

**Scheme 9:**
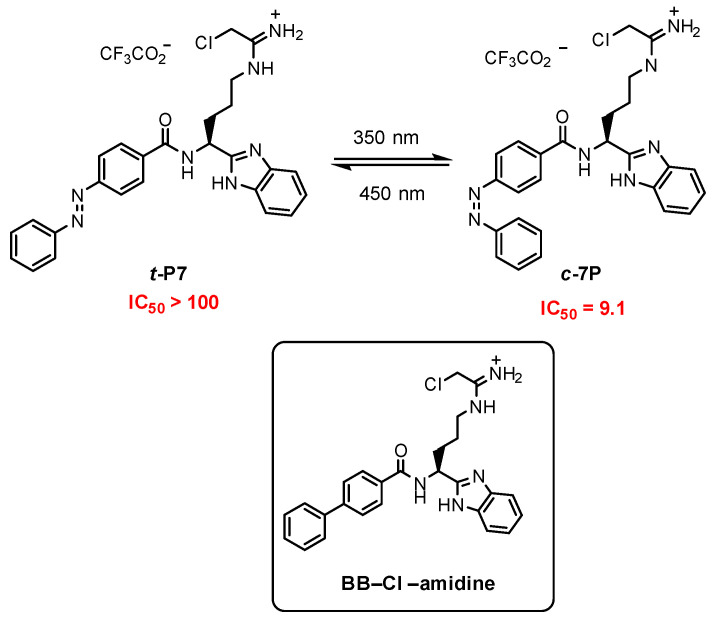
Photoisomerization of **P7** and the corresponding parent inhibitor.

Protein arginine deiminases (PADs) are cysteine hydrolases that mediate the conversion of arginine to citrulline [73]. Mondal and co-workers [74] developed a series of *cis*-on photoswitchable inhibitors for PAD2. Among them, **P7** (Scheme 9) is the most promising candidate for the inhibition of PAD2. **P7** was designed by modification of a second-generation PAD inhibitor, BB-Cl-amidine [75,76], with an azobenzene unit. Photoisomerization of ***t*****-P7** and ***c*****-P7** could be achieved by irradiation with 350 nm and 450 nm light, respectively, and more than 80% of ***c*-P7** was obtained at PPS_350nm_.

The inhibition of ***t*-P7** and ***c*-P7** to PAD2 was performed by the competitive activity-based protein profiling (ABPP) assay. It was found that the potency was increased by 10-fold upon excitation to the ***c*-P7**. The IC_50_ value of ***t*-P7** is >100 μM, whereas the IC_50_ value of ***c*-P7** is 9.1 μM. The increased potency upon photoisomerization is most likely due to enhanced binding to the PAD2 active site.

**Figure 4:**
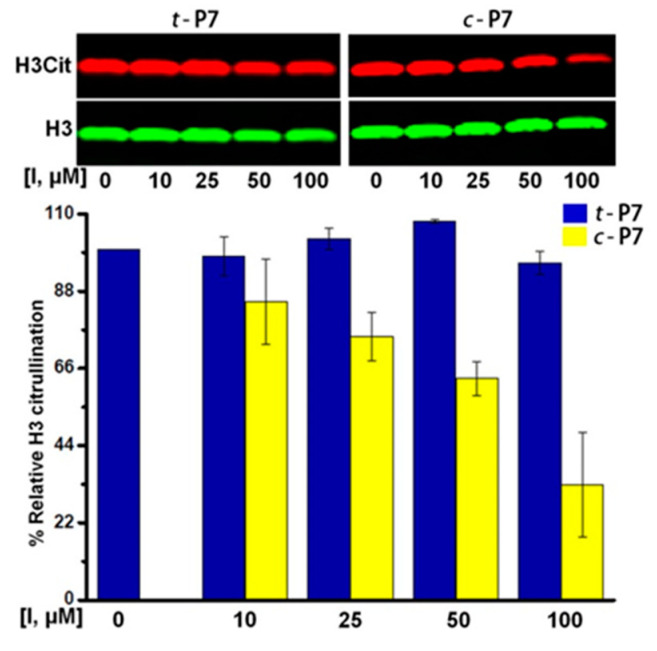
Inhibition of histone H3 citrullination in HEK293T/PAD2 cells by **P7**. Inhibitor concentrations [I] are given under each lane of the Western blot image. Citrullinated H3 (H3 Cit) and H3 are shown in red and green, respectively. The quantification of each band yielded the H3 Cit/H3 ratio, from which the % relative H3 citrullination was calculated. Reproduced with permission from [74]. Copyright 2018 American Chemical Society.

The inhibition of **P7** to PAD2 in cells was performed by evaluation the ability of **P7** to inhibit histone H3 citrullination in HEK293T/PAD2-overexpressing cells [77,78]. ***t*-P7** exhibited no inhibition to histone H3 citrullination even at 100 μM, while ***c*-P7** inhibited citrullination in a dose-dependent manner (Figure 4), which suggested that **P7** could be photoactivated to inhibit histone H3 citrullination in HEK293T/PAD2 cells.

**Scheme 10:**
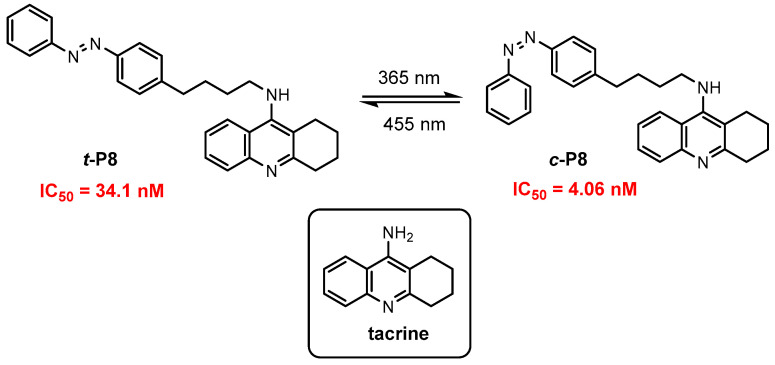
Photoisomerization of **P8** and the corresponding parent inhibitor.

Scheiner and co-workers [79] constructed a class of photoswitchable inhibitors for the enzyme acetylcholinesterase (AChE); among which, **P8** exhibited the most outstanding performance. **P8** (Scheme 10) was constructed by combining a known AChE inhibitor tacrine [80,81] and azobenzene with a C4 aliphatic alkyl chain. A binding model of ***t*-P8** in a complex with tcAChE [82] showed that the linker length of the photoswitchable moiety to tacrine has influence not only on the biological interaction but also on the physicochemical properties. Photoisomerization of **P8** could be achieved by irradiation at 365 nm and 455 nm, respectively, and 63% of ***c*-P8** at PPS_365nm_ and 80% of ***t*-P8** at PPS_455nm_ were obtained.

The inhibitory activity of both ***c*-P8** and ***t*-P8** against hAChE was evaluated, in which an 8.4-fold increase in activity was observed with **c-P8** (IC_50_ = 4.06 nM, ***t*-P8**: IC_50_ = 34.1 nM). Additionally, both isomers showed selectivity towards hAChE, but ***c*-P8** had better selectivity with a factor of 70 than ***t*-P8** with a factor of 19.

**Scheme 11:**
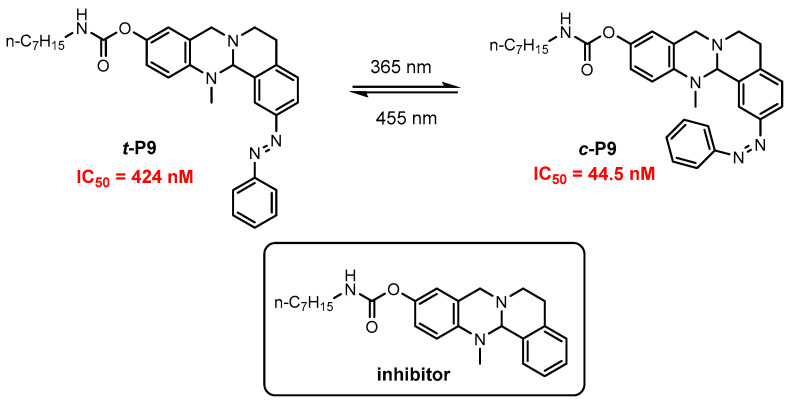
Photoisomerization of **P9** and the corresponding parent inhibitor.

For insight into Alzheimer’s disease, the same research group [83] has recently prepared a class of photoswitchable butyrylcholinesterase (BChE) inhibitors and applied them to Alzheimer’s disease in mouse model. **P9** (Scheme 11) was prepared by conjugation of a hBChE inhibitor [84,85] with azobenzene. Photoisomerization of **P9** was carried out by irradiation at 365 nm and 455 nm, respectively, and 81% **c-P9** at PPS_365nm_ and 82% ***t*-P9** at PPS_455nm_ were obtained.

The inhibition of **P9** to hBChE was in the nanomolar range and showed a 10-fold difference in the IC_50_ values between **c-P9** (IC_50_ = 44.5 nM) and **t-P9** (IC_50_ = 424 nM). Docking studies explained the differences of the two isomers in the association step of the carbamylation and the less favorable Kc of ***t*-P9** over ***c*-P9** and suggested that ***t*-P9** is unable to conformationally adapt to the binding pocket.

**Figure 5:**
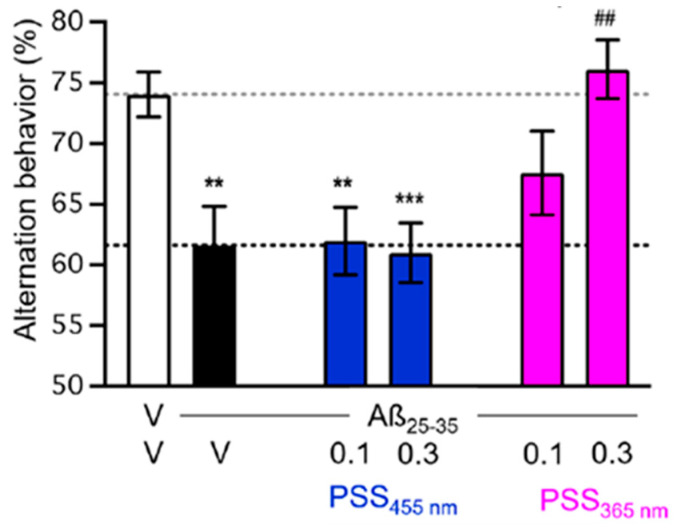
Effects of both **t-P9** (PSS_455nm_) and **c-P9** (PSS_365nm_) on Aβ_25–35_-induced spontaneous alternation deficits with an anti-amnesic murine AD model. Mice received Aβ_25–35_ (9 nmol icv) or vehicle solution (3 μL icv) on day 1 and inhibitor **P9** in the 0.3–1 mg/kg ip dose range 30 min before the YMT session on day 8. Data show means ± SEM of n = 12 per group. ANOVA: F(5,64) = 55.937, *p* = 0.0001. ** *p* < 0.01, and *** *p* < 0.001 vs. (V/V)-treated group and ## *p* < 0.01 vs. (Aβ_25–35_/V)-treated group, Dunnett’s post hoc test. Aβ_25–35_, amyloid-β_25–35_; icv, intracerebroventricular; ip, intraperitoneal; V, vehicle. Reproduced with permission from [83]. Copyright 2022 American Chemical Society.

It is reported that prolonged duration of the inhibition of BChE directly correlates to neuroprotective effects in vivo upon chronic administration [86]. Inhibitor **P9** was administered intraperitoneally into mice to study the difference in an anti-amnesic model in vivo. As shown in Figure 5, ***c*-P9** was effective in the 0.1–0.3 mg/kg dose range in attenuating the Aβ_25–35_-induced alternation deficit. ***c*-P9** allowed a complete recovery at a dosage of 0.3 mg/kg, whereas ***t*-P9** showed no effect at all.

**Scheme 12:**
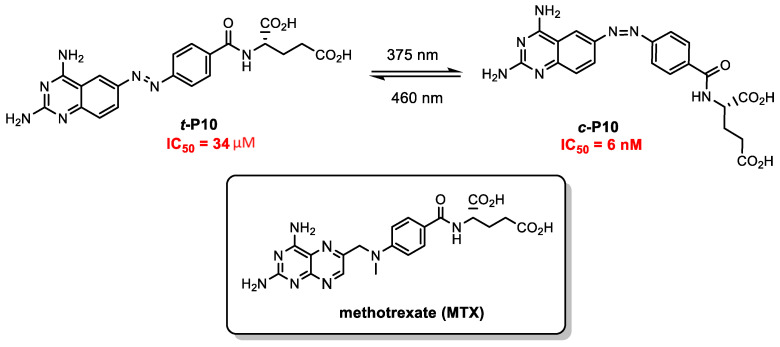
Photoisomerization of **P10** and the corresponding parent inhibitor.

Matera and co-workers [87] designed a photoswitchable inhibitor for photoregulating the human dihydrofolate reductase (DHFR). **P10** (Scheme 12) was obtained by modification of the inhibitor methotrexate (MTX) [88,89] with an azo unit. Docking studies showed that ***c*-P10** bound to the active site of the target enzyme in a mode that strictly mimics the orientation and conformation adopted by MTX in its crystallographic pose. In contrast, ***t*-P10** displayed a set of binding poses that barely overlapped with MTX. Photoisomerization of *cis*- and *trans*-**P10** could be achieved by irradiation with 375 nm and 460 nm, respectively, and 75% of ***c*-P10** was obtained at PPS_375 nm_.

**Figure 6:**
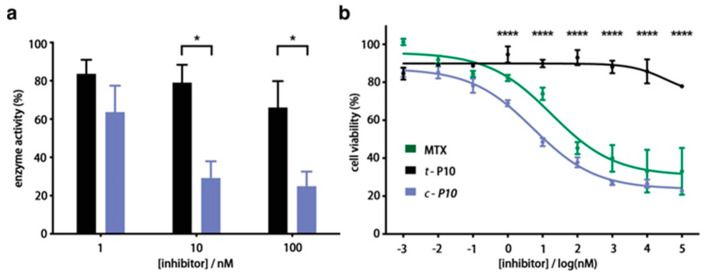
(**a**) Inhibition of DHFR activity at 1 nM, 10 nM, and 100 nM of ***t*-P10** (black) and ***c*-P10** (violet). Data are means of at least three independent experiments in triplicate or quadruplicate ± SEM. Adjusted *p*–value (*) ≤ 0.05. (**b**) HeLa cell viability assay at different concentrations of MTX, ***c*-P10**, and ***t*-P10**. Data are means of at least three independent experiments in triplicate or quadruplicate ± SEM. Adjusted *p*-value (****) ≤ 0.0001. Reproduced with permission from [87]. Copyright 2018 American Chemical Society.

Inhibition of two isomers to DHFR was assessed firstly by the investigation of **P10** to inhibit the purified target enzyme using a colorimetric assay. MTX was tested as the positive control. As shown in Figure 6a, significant differences between the two isomers were observed. ***cis*-P10** exhibited much stronger inhibiting DHFR activity than ***t*-P10** at the same concentration. A remarkable difference in IC_50_ values determined for ***c*-P10** (6 nM) and ***t*-P10** (34 µM) was obtained. Similar results were observed in cells. As shown in Figure 6b, ***c*-P10** exhibited significantly lower viability in HeLa cells than ***t*-P10**.

**Figure 7:**
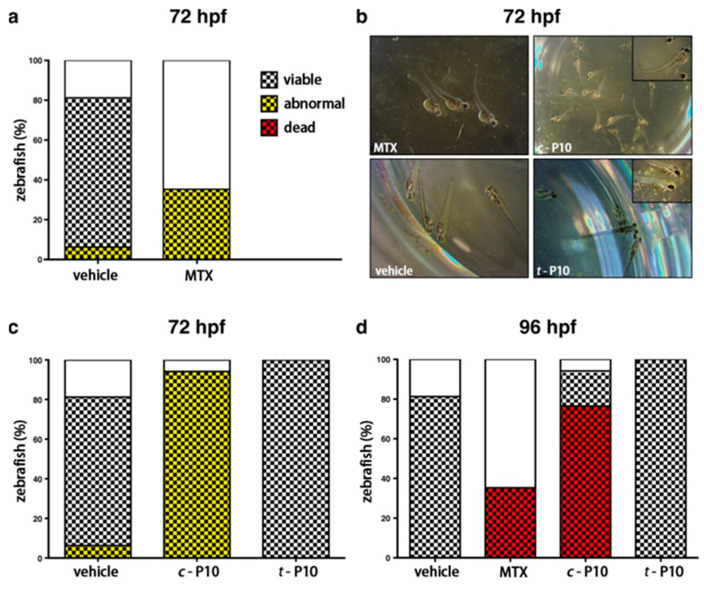
Effects of ***t*-P10** and ***c*-P10** (obtained by external pre-irradiation with 375 nm light) on zebrafish development, viability, and mortality. (**a**,**c**) Anatomical profiles of zebrafish at 72 hpf after treatment with vehicle (DMSO 2%), MTX (200 μM), ***c*-P10** (200 μM), and ***t*-P10** (200 μM). The graph shows the percentage of zebrafish treated vs. the treatment group, superimposing the number of abnormal larvae dead zebrafish (black and yellow checkered sections) onto the number of viable embryos (checkered sections) and onto the total number of fertilized embryos (whole columns). (**b**) Illustrative pictures of individual larvae from each treatment group at 72 hpf. (**d**) Mortality of zebrafish at 96 hpf after treatment with vehicle (DMSO 2%), MTX (200 μM), ***c*-P10** (200 μM), and ***t*-P10** (200 μM). The graph shows the percentage of zebrafish treated vs. the treatment group, superimposing the number of dead zebrafish (black and red checkered sections) onto the number of viable embryos (checkered sections) and onto the total number of fertilized embryos (whole columns). Reproduced with permission from [87]. Copyright 2018 American Chemical Society.

In vivo evaluation of **P10** to DHFR was performed in zebrafish. Since DHFR inhibitors disrupt folate metabolism, they have a high impact at the early stages of animal development. Zebrafish fertilized eggs were incubated within 5 h post-fertilization (hpf) in UV-purified water containing ***t*-P10** or ***c*-P10**. Embryos treated with MTX showed a low viability (Figure 7a). Three abnormalities (deficient iridiophore ocular pigmentation, an abnormal volume of the cardiac cavity, and tail angle deviations) were observed in MTX-treated, ***t*-P10**-treated, and ***c*-P10**-treated zebrafishes (Figure 7b). ***c*-P10** exhibited high toxicity and produced comparable developmental abnormalities at 72 hpf, together with a high rate of mortality at 96 hpf. In contrast, ***t*-P10** showed a low toxicity, and no observable abnormality at 72 hpf and no mortality at 96 hpf were observed (Figure 7c).

Inhibitor **P10** was also employed by Mashita and co-workers [90] for photoregulating *Escherichia coli* dihydrofolate reductase (eDHFR) activity. The IC_50_ values determined for ***t*-10** and ***c*-P10** were 45 ± 4 nM and 3.2 ± 0.4 nM, respectively.

**Scheme 13:**
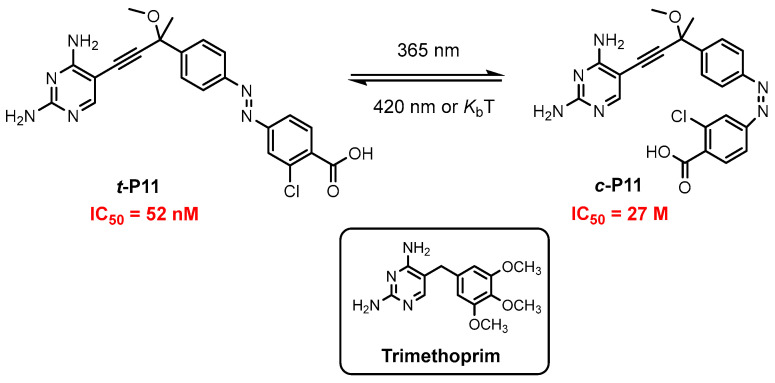
Photoisomerization of **P11** and the corresponding parent inhibitor.

Kobauri and co-workers [91] evaluated different structure-based approaches for photopharmacology with eDHFR as a case study. **P11** (Scheme 13), a representative of a series of synthesized compounds, was obtained by the modification of trimethoprim, one of the eDHFR inhibitors [92,93] with an azobenzene unit.

Photoisomerization of ***t*-P11** and ***c*-P11** was carried out with 365 nm and 420 nm/in the dark, respectively, and 79% of ***c*-P11** at PPS_365nm_ and 89% of ***t*****-P11** at PPS_420nm_ were obtained. The light-dependent potency showed that both isomers exhibited strong inhibition, with a two-fold increase in IC_50_ when ***c*-P11** was irradiated with 365 nm light.

**Scheme 14:**
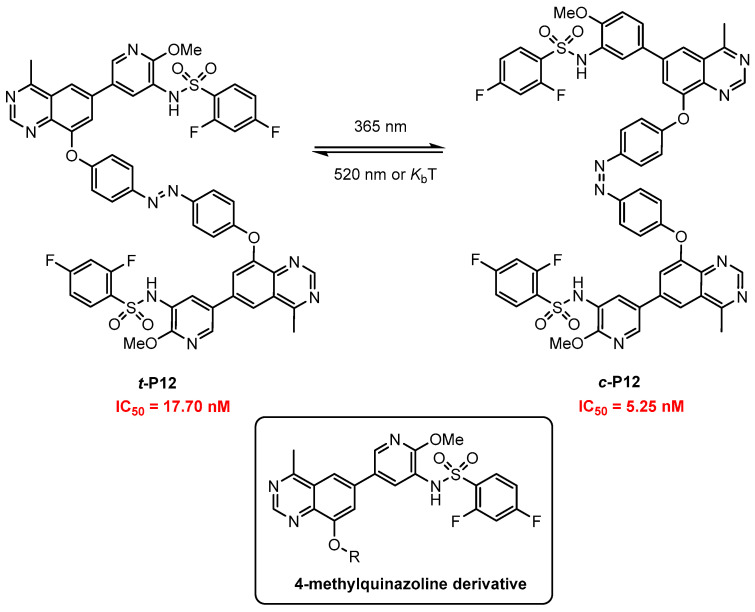
Photoisomerization of **P12** and the corresponding parent inhibitor.

The phosphoinositide 3-kinase (PI3K) signaling pathway is essential for regulating various cellular processes, and the dysregulation of PI3K leads to human cancers [94]. Zhang and co-workers [95] developed a photoswitchable PI3K inhibitor based on a 4-methylquinazoline derivative [96,97]. **P12** (Scheme 14), synthesized by conjugating the 4-methylquinazoline derivative and azobenzene, exhibited photoisomerization of *cis*- and *trans*-**P12** upon irradiation with 365 nm and 520 nm, respectively, and 90% of ***c*-P12** was obtained at PPS_365nm_.

A PI3Kα activity inhibition assay showed that ***c*-P12** exhibited stronger inhibition than ***t*-P12** at the same concentration, and approximately three-fold more potency was observed for the ***c*-P12** (IC_50_ = 5.24 nM) compared to ***t*****-P12** (IC_50_ = 17.70 nM). Cellular photo-regulating of PI3K activity was evaluated with **P12** performed in HGC-27 cells that harbor the PI3KCA mutation and have an overactive PI3K pathway. Western blotting analysis showed that both ***t*-P12** and ***c*-P12** inhibited the phosphorylation of AKT (p-AKT, S473, and T308) and S6 in a dose-dependent manner. ***c*-P12** suppressed p-AKT (S473 and T308) and S6 more potently than ***t*-P12**. The activity transformation could be achieved with photoswitchable **P12** upon 365 nm and 520 nm alternating irradiation.

**Figure 8:**
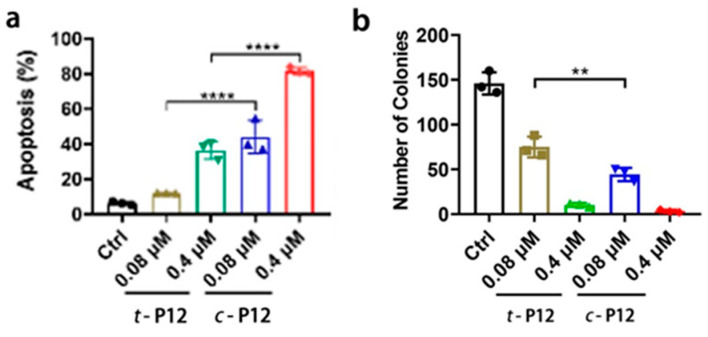
(**a**) Apoptosis analysis of HGC-27 cells by flow cytometry with ***t*-P12** and ***c*-P12**. Results are expressed as the mean ± SEM (n = 3 for each group), one-way ANOVA analysis, **** *p* < 0.0001 between indicated groups. (**b**) Colony formation assay of HGC-27 cells with ***t*-P12** and ***c*-P12**, ** *p* < 0.01 between indicated groups. Reproduced with permission from [95]. Copyright 2022 Elsevier.

Apoptosis induced by **P12** was detected by flow cytometry using Annexin V as a probe. As shown in Figure 8a, both ***t*-P12** and ***c*-P12** dose-dependently promoted apoptosis in HGC-27 cells. The apoptotic ratios of the ***t*-P12**-treated group were significantly lower than those of the ***c*-P12** at concentrations of 0.08 µM and 0.04 μM. Furthermore, the colony formation assay exhibited that both ***t*-P12** and ***c*-P12** reduced the number of colonies significantly in a dose-dependent manner (Figure 8b). Compared to ***t*-P12**, ***c*-P12** showed stronger inhibitory activity against HGC-27 colony formation.

## 4. Conclusions and Outlook

Photopharmacology endows therapeutics with light addressability; this, in turn, allows for improved spatial and temporal selectivity in drug action, which provides a new concept and strategy for therapy [98,99]. The idea of photopharmacology was demonstrated as early as 1969 [100], but it is only recently that photopharmacology has shown its potential in future therapeutic applications. Photoregulating activity has been demonstrated in a range of live cell and animal models [101,102,103], and translationally promising results have been reported in the area of vision restoration [104,105,106]. 

Currently, photoswitchable agents for photopharmacology are mainly based on organic photochromic compounds. In addition to azobenzenes [107,108], photochromic spiropyrans [109], fulgimides [110], and diarylethenes [111] are also employed as photoswitches for the photomodulation of biological properties. These systems have their own advantages and disadvantages, for example, azobenzenes and spiropyrans are easily obtained and have large conformation changes after irradiation but show low photochemical reversibility and poor thermal stability; fulgide/fulgimides and diarylethenes show high photochemical reversibility and good thermal stability but small conformation change and difficult synthesis. Therefore, the development of novel photoswitches, as well as their convenient synthesis methods, is a key task in this field [112,113,114].

In this review, recent advances in azobenzene-based photoswitchable inhibitors for regulating enzyme activity have been summarized. As an optical switching unit, azobenzenes are the largest and most-studied photoswitching molecules in biology [115,116,117,118,119] because of easy accessibility and the large conformation change before and after irradiation. However, the deficiency of azobenzene-based photoswitchable agents is also obvious, as mentioned above. In addition, a short absorption wavelength (≤600 nm) is also a key factor limiting its practical use due to insufficient penetration depth. Major developments in the design of photoswitchable inhibitors are needed to fulfill all the prerequisites for pharmacotherapy [120,121,122,123]. In particular, photoswitches could be photoisomerized in both directions with light in the therapeutic window (650–1200 nm) with good thermal stability of both isomers. To obtain the near-infrared (NIR) irradiation wavelength, two/multiphoton absorption photoswitchable agents are desired [124,125,126]. The quantum yield of photoisomerization should be as high as possible, and the difference in the affinity for the target between the active and inactive form must be significant (>100-fold). Other requirements in the design of photoswitchable inhibitors include water solubility, low toxicity, metabolic stability, and not interfering with the activity of other biological molecules. It is a long and winding road from the design of photoswitchable agents to clinical application, but with more and more study and outcomes [127,128,129,130,131], it could be expected that this area will make further progress and be applied in clinics in the future.

## Data Availability

Not applicable.
